# Supplementary material for: Growth rate-driven modelling suggests that phenotypic adaptation drives drug resistance in BRAFV600E-mutant melanoma
Source: Commun Biol. 2026 Feb 26;9:385. doi: 10.1038/s42003-026-09760-2 (PMC12992687; doi:10.1038/s42003-026-09760-2)
Supplement: Supplementary file 2 — Supplementary Information [file 42003_2026_9760_MOESM2_ESM.pdf]

Supplementary Material to: Growth rate-driven modelling suggests that phenotypic adaptation drives drug resistance in BRAFV600E-mutant melanoma

Sara Hamis[1⊛], Alexander P Browning[2], Adrianne L Jenner[3], Chiara Villa[4,5], Philip K Maini[2], Tyler Cassidy[6].

1 Department of Information Technology, Uppsala University, Uppsala, Sweden.
2 Mathematical Institute, University of Oxford, Oxford, United Kingdom.
3 School of Mathematical Sciences, Queensland University of Technology, Brisbane, Australia.
4 Sorbonne Université, CNRS, Université de Paris, Inria, Laboratoire Jacques-Louis Lions UMR, Paris, France.
5 Université Paris-Saclay, Inria, Centre Inria de Saclay, 91120, Palaiseau, France.
6 School of Mathematics, University of Leeds, Leeds, United Kingdom.

Table of contents

SM1: Parameter fitting and complementary simulation results (page 1)
SM2: Expressions for effective growth rates under intermittent treatments (page 6)
SM3: Model parameters used in main the article results figures (page 12)

SM1: Parameter fitting and complementary simulation results

In Figure 2d of the main manuscript, the treatment response models include four parameters that are fitted to match data. These are: $\text{FM}(0, 500\text{ nM}), \text{FM}(1, 500\text{ nM}), \eta_{\text{on}}, \eta_{\text{off}}$. As an alternative approach, one could consider fixing $\text{FM}(0, 500\text{ nM}), \text{FM}(1, 500\text{ nM})$ via log-linear interpolation (the open circles in Supplementary Figure 1), and thereafter fitting only $\eta_{\text{on}}, \eta_{\text{off}}$. However, with this approach, we were not able to find model parameters that yield simulations which simultaneously match both continuous and intermittent treatment responses. This result is shown in Supplementary Figure 2, which also demonstrates that for all update models, save the biased one, the 300 nM model and the 500 nM model with four free parameters result in similar and often overlapping simulated treatment dynamics.

In Supplementary Figures 3 to 6 we have reconstructed Figure 2d (with four free parameters) for different values of n, i.e., the number of intermittent phenotypes between the extrema $x = 0$ and $x = 1$, and ρ, i.e., the probability that a cell accepts a proposed phenotype update. The results show that the directed (semi-biased and biased) update strategies better capture the cell count dynamics in response to treatments than do the non-directed (no update and unbiased) update strategies for tried values of $n > 0$, given the fitness matrix with linearly interpolated growth rates between the fitted extrema $\text{FM}(0, 500\text{ nM}), \text{FM}(1, 500\text{ nM})$.

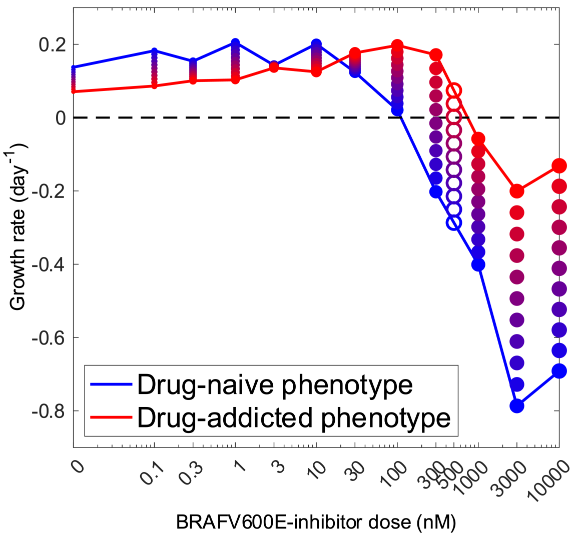

Supplementary Figure 1: 500 nM growth rates (open circles) can be estimated through log-linear interpolation between 300 nM and 1000 nM growth rates.

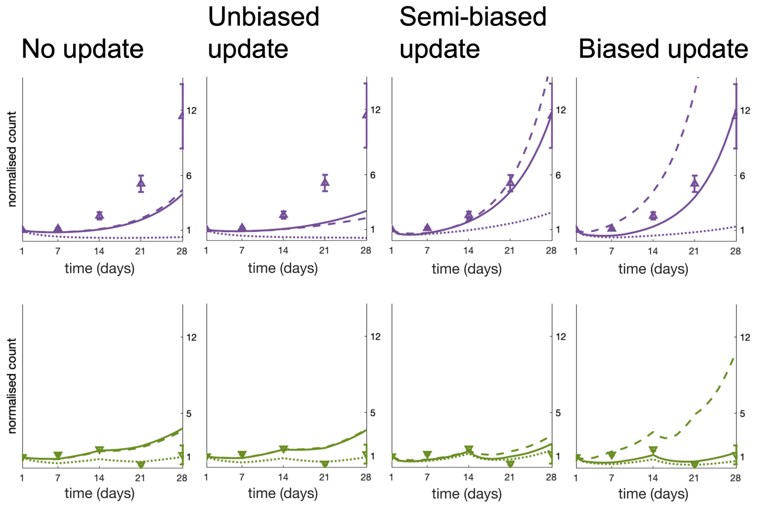

Continous treatment

In vitro data.

Simulated responses to 300 nM with $\eta_{on} = \eta_{off} = 2$.

Simulated responses to 500 nM with fitted FM(0,500), FM(1,500), η_{on}, η_{off}.

Simulated responses to 500 nM with interpolated FM(0,500), FM(1,500) and fitted η_{on}, η_{off}.

Intermittent treatment

In vitro data.

Simulated responses to 300 nM with $\eta_{on} = \eta_{off} = 2$.

Simulated responses to 500 nM with fitted FM(0,500), FM(1,500), η_{on}, η_{off}.

Simulated responses to 500 nM with interpolated FM(0,500), FM(1,500) and fitted η_{on}, η_{off}.

Supplementary Figure 2: Simulated responses to continuous (top) and intermittent (bottom) treatments are shown for different phenotype update strategies (left-to-right) and three different model parameter combinations (see legend). In vitro data (triangles) are also shown.

1

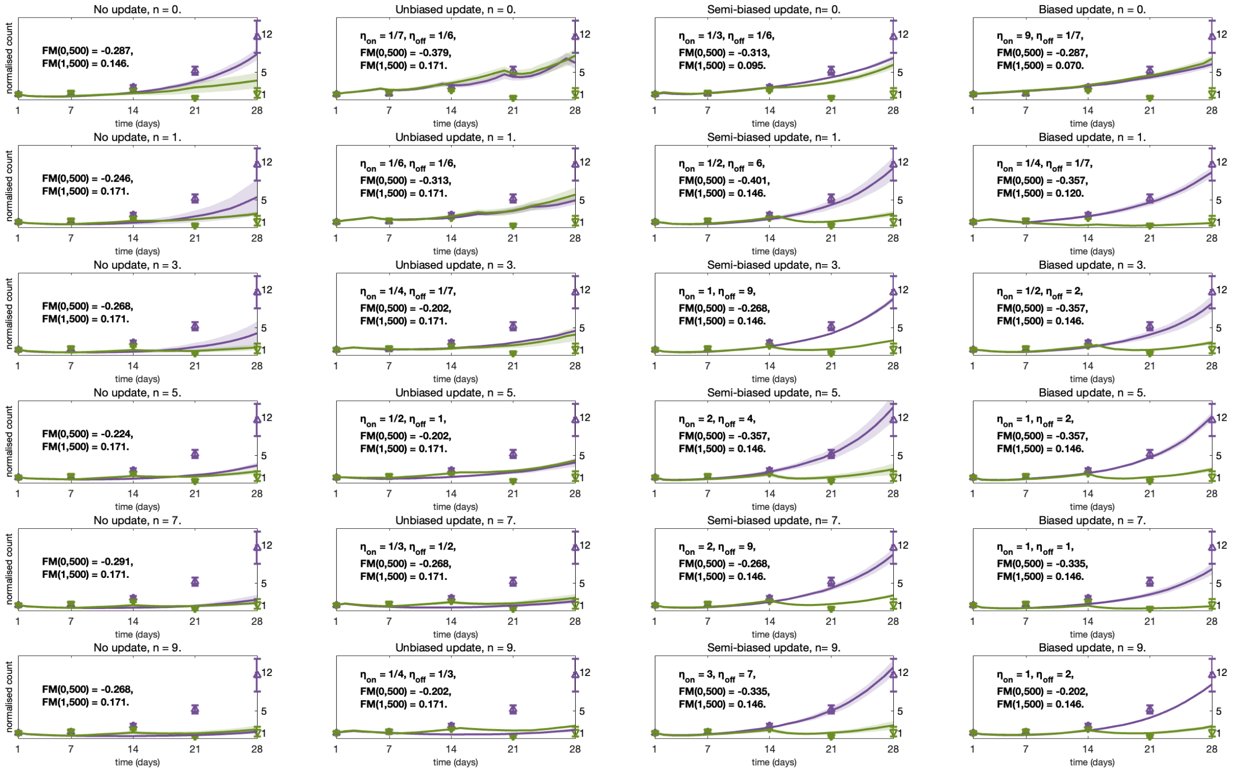

Supplementary Figure 3: **Simulated treatment responses for $\rho = 1$.** Plots are shown for different phenotype update strategies (left to right), and for varying numbers of intermediate phenotypes n between the extrema $x = 0$ and $x = 1$. The number of phenotypes n increases as we go from higher to lower rows. In each plot, the model parameters η_{on}, η_{off}, FM($x = 0$,$d = 500$), FM($x = 1$,$d = 500$) that simultaneously minimise the root mean squared error between data and simulations for both continuous (purple) and intermittent (green) treatment responses to 500 nM doses are used.

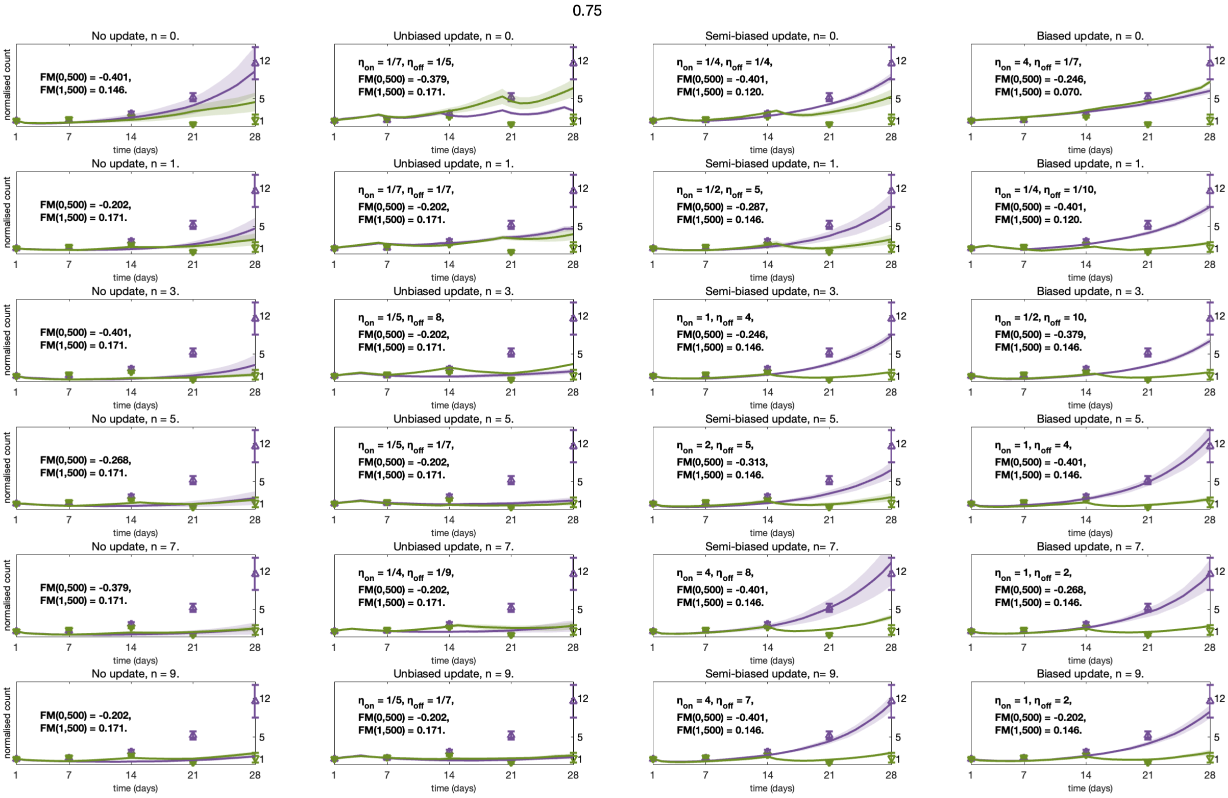

Supplementary Figure 4: **Simulated treatment responses for $\rho = 0.75$.** Plots are shown for different phenotype update strategies (left to right), and for varying numbers of intermediate phenotypes n between the extrema $x = 0$ and $x = 1$. The number of phenotypes n increases as we go from higher to lower rows. In each plot, the model parameters η_{on}, η_{off}, FM($x = 0$,$d = 500$), FM($x = 1$,$d = 500$) that simultaneously minimise the root mean squared error between data and simulations for both continuous (purple) and intermittent (green) treatment responses to 500 nM doses are used.

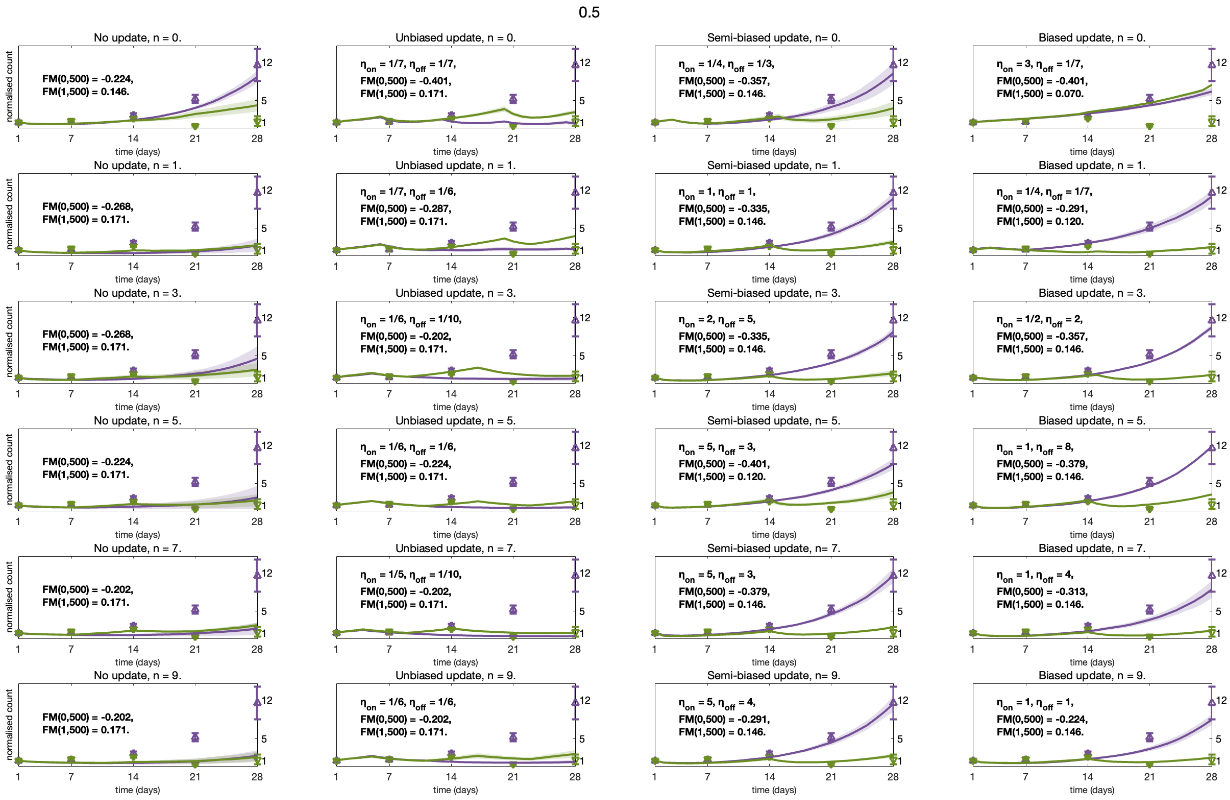

Supplementary Figure 5: **Simulated treatment responses for** $\rho = 0.5$**.** Plots are shown for different phenotype update strategies (left to right), and for varying numbers of intermediate phenotypes n between the extrema $x = 0$ and $x = 1$. The number of phenotypes n increases as we go from higher to lower rows. In each plot, the model parameters η_{on}, η_{off}, FM($x = 0$,$d = 500$), FM($x = 1$,$d = 500$) that simultaneously minimise the root mean squared error between data and simulations for both continuous (purple) and intermittent (green) treatment responses to 500 nM doses are used.

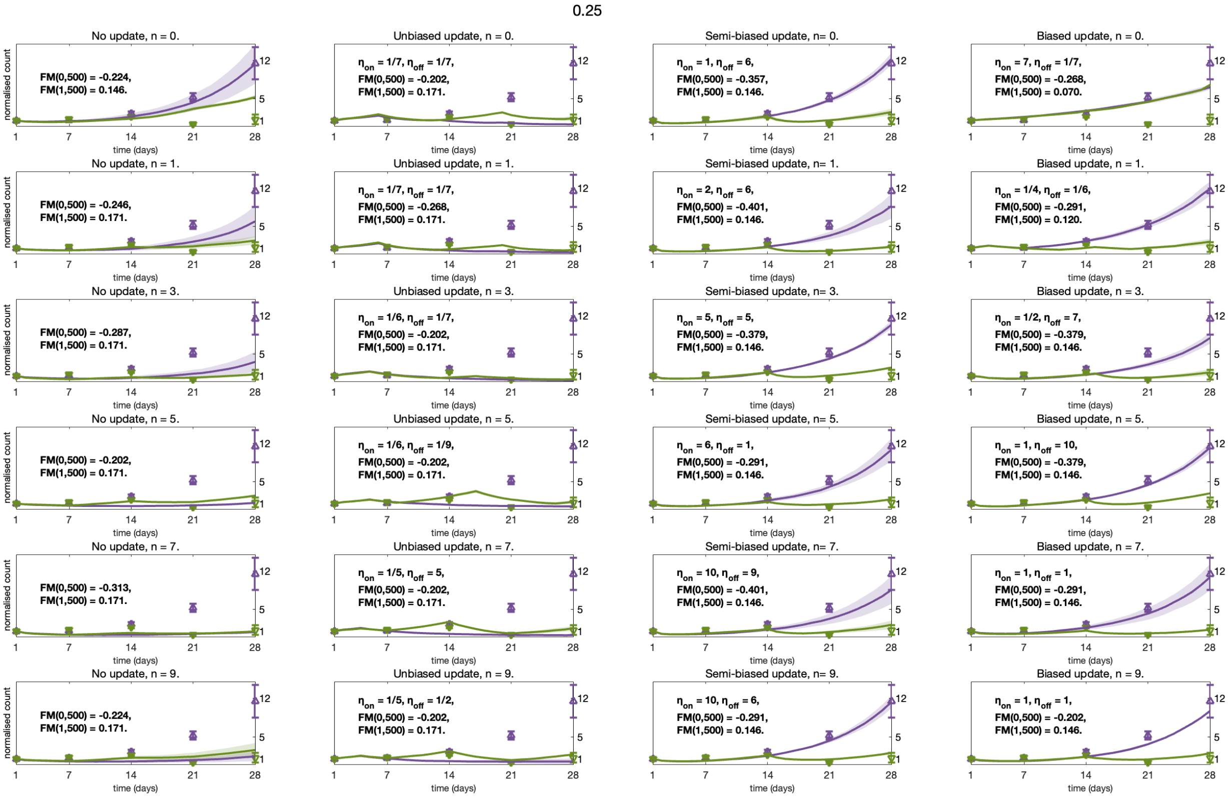

Supplementary Figure 6: **Simulated treatment responses for $\rho = 0.25$.** Plots are shown for different phenotype update strategies (left to right), and for varying numbers of intermediate phenotypes n between the extrema $x = 0$ and $x = 1$. The number of phenotypes n increases as we go from higher to lower rows. In each plot, the model parameters η_{on}, η_{off}, FM($x = 0$,$d = 500$), FM($x = 1$,$d = 500$) that simultaneously minimise the root mean squared error between data and simulations for both continuous (purple) and intermittent (green) treatment responses to 500 nM doses are used.

SM2: Expressions for effective growth rates under intermittent treatments

A Wolfram Mathematica notebook for calculating long-term effective growth rates for intermittent treatment schedules (Eq. 6 in the main article) are available on the GitHub repository `https://github.com/SJHamis/phenotype_adaptation`. A printed version of the notebook is provided on the next few pages.

Growth-rate driven modelling study

Supplementary material to *Hamis, Browning, Jenner, Villa, Maini, Cassidy (2025).*

In this Notebook we calculate
$\lambda_{\text{eff}} = \frac{1}{\tau}\left(\int_0^{t_{on}} FM(x(t), d)\,dt + \int_{t_{on}}^{t_{off}} FM(x(t), 0)\,dt\right)$
for different choices of t_{on} (the duration that the drug is *on*) and t_{off} (the duration that the drug is *off*).

Define growth rates as functions of the phenotype state x:

In the notebook, FMxd denotes FM(x,d) where
x=0 is the least drug-adapted phenotype state,
x=1 is the most drug adapted phenotype state,
d=0 means “drug is off”,
d=D means “drug is on” at a selected dose D.

In[1]:=
```
λ0[x_] := FM00 + (FM10 - FM00) x (* no drug *)
λ1[x_] := FM0D + (FM1D - FM0D) x (* drug *)
```

State assumptions:

In[3]:=
```
$Assumptions = ω > 0;
```

Case 1: $t_{on}, t_{off} > \frac{1}{\omega}$.

Drug on: Phenotypes start at x=0, reach x=1 during climbing period of length $1/\omega$, and then stay at x=1 until time t_{on}.
Drug off: Cells reach x=0 during a descending period of length $1/\omega$, and then stay at x=0 until time $t_{on}+t_{off}$.
A plot is shown below for (arbitrarily selected) values of t_{on}, t_{off}, ω such that Case 1 holds.

In[4]:=
```
x[t_] := Piecewise[{{ω t, t < 1/ω}, {1, 1/ω ≤ t ≤ ton},
    {1 - ω (t - ton), ton < t < ton + 1/ω}, {0, t ≥ ton + 1/ω}}];
Plot[x[t] /. {ω → 0.5, ton → 5, toff → 10}, {t, 0, 15}, AxesLabel → {"t", "x(t)"}]
```

Out[5]=

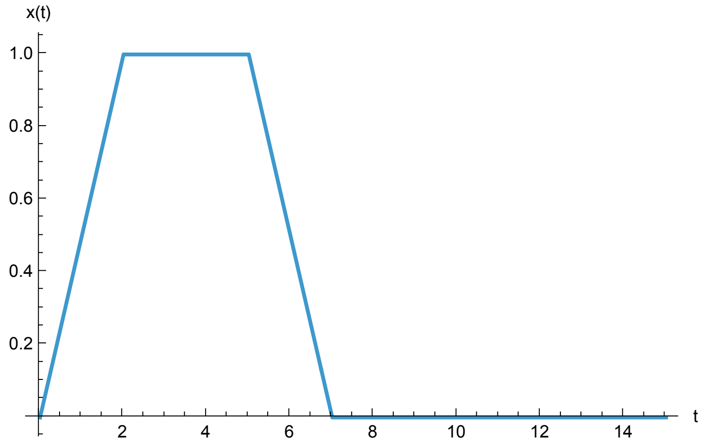

We now symbolically calculate and simplify the expression for the Case 1 integral using $\Delta\text{FM}(d) = \text{FM}(1, d) - \text{FM}(0, d)$.

In[6]:=
```
C1 = 1/τ FullSimplify[
     Integrate[λ1[ω t], {t, 0, 1/ω}] +
     Integrate[λ1[1], {t, 1/ω, ton}] +
     Integrate[λ0[1 - ω (t - ton)], {t, ton, ton + 1/ω}] +
     Integrate[λ0[0], {t, ton + 1/ω, ton + toff}]
   ] /. {FM10 - FM00 → ΔFM0} /. {FM0D - FM1D → -ΔFMD}
```

Out[6]=
$$\frac{\text{FM00 toff} + \text{FM1D ton} + \frac{\Delta\text{FM0} - \Delta\text{FMD}}{2\,\omega}}{\tau}$$

Now customised, numerical values for t_{on}, t_{off}, ω, can be inserted into the expression for C1 to obtain λ_{eff} .

In[7]:=
```
C1num = C1 /. {ΔFM0 → FM10 - FM00, ΔFMD → FM1D - FM0D, τ → ton + toff};
(*Writing C1 in long format*)
C1num /. {FM0D → -0.2866, FM1D → 0.0741, FM00 → 0.1374, FM10 → 0.07067,
   ω → 0.5, ton → 5, toff → 10} (*As used in the above plot*)
```

Out[8]= 0.0878047

Case 2: $t_{on} < t_{off} < \frac{1}{\omega}$.

Drug on: Phenotypes start at x=0, and then reach x=ωt during climbing period of length t_{on}.
Drug off: Cells reach x=0 during a descending period of length t_{on}, and then stay at x=0 until time t_{on}+ t_{off}.

A plot is shown below for (arbitrarily selected) values of t_{on}, t_{off}, ω such that Case 2 holds.

In[9]:=
```
x[t_] := Piecewise[
  {{ω t, t < ton}, {ω ton - ω (t - ton), ton ≤ t < 2 ton}, {0, 2 ton ≤ t ≤ ton + toff}}]
Plot[x[t] /. {ω → 0.1, ton → 3, toff → 10}, {t, 0, 15}]
```

Out[10]=

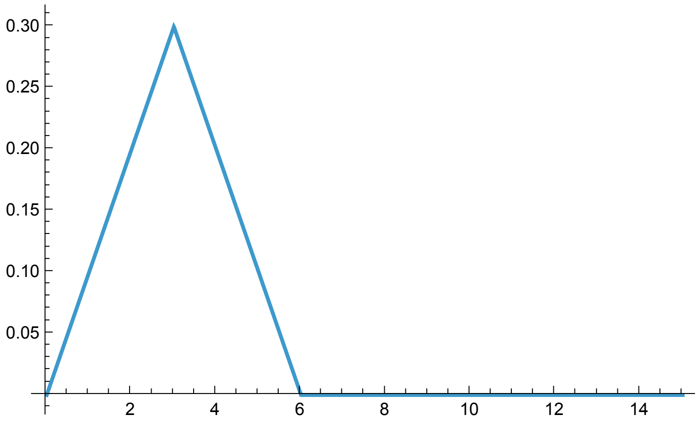

We now symbolically calculate and simplify the expression for the Case 2 integral using $\Delta FM(d) = FM(1, d) - FM(0, d)$.

In[11]:=
```
C2 = 1/τ FullSimplify[
    Integrate[λ1[ω t], {t, 0, ton}] +
    Integrate[λ0[ω ton - ω (t - ton)], {t, ton, 2 ton}] +
    Integrate[λ0[0], {t, 2 ton, ton + toff}]
  ] /. {FM10 - FM00 → ΔFM0} /. {-FM0D + FM1D → ΔFM1}
```

Out[11]=

$$\frac{\text{FM00 toff} + \text{FM0D ton} + \frac{1}{2}\,\text{ton}^2\,(\Delta\text{FM0} + \Delta\text{FM1})\,\omega}{\tau}$$

Case 3: $t_{off} < t_{on} < \frac{1}{\omega}$.

Drug off: Phenotypes start at x=1, and then reach x=ωt during descending period of length t_{off}.
Drug on: Phenotypes reach x=1 during climbing period of length t_{off}, and then stay at x=1 until time t_{on}+ t_{off}.
A plot is shown below for (arbitrarily selected) values of t_{on}, t_{off}, ω such that Case 2 holds.

In[12]:=
```
x[t_] := Piecewise[{{1 - ω t , t < toff},
    {1 - ω toff + ω (t - toff), toff ≤ t < 2 toff}, {1, 2 toff ≤ t ≤ ton + toff}}]
Plot[x[t] /. {ω → 0.1, ton → 10, toff → 3}, {t, 0, 15}]
```

Out[13]=

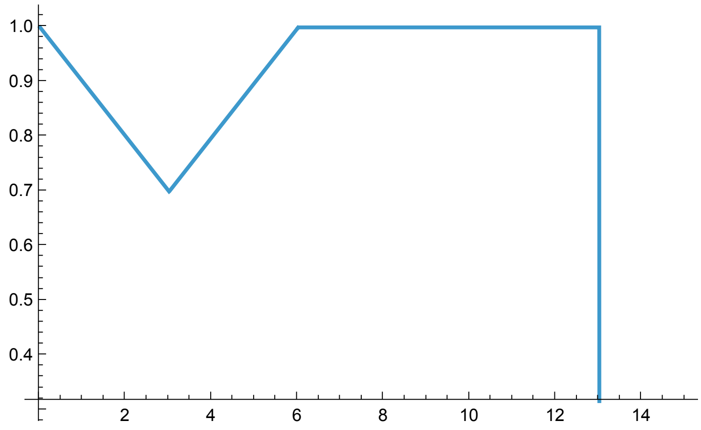

In[14]:=
```
C3 = 1/T FullSimplify[
    Integrate[λ0[1 - ω t], {t, 0, toff}] +
    Integrate[λ1[1 - ω toff + ω (t - toff)], {t, toff, 2 toff}] +
    Integrate[λ1[1], {t, 2 toff, ton + toff}]
  ] /. {FM0D - FM1D → -ΔFMD}  /. {FM00 - FM10 → -ΔFM0}
```

Out[14]=

$$\frac{\text{FM10 toff} + \text{FM1D ton} + \frac{1}{2}\,\text{toff}^2\,(-\Delta\text{FM0} - \Delta\text{FMD})\,\omega}{T}$$

Case 4: $t_{on} = t_{off} = \frac{T}{2} = \frac{1}{\omega}$.

Drug on: Phenotypes start at x=0, and then reach x=1 during climbing period of length $\frac{T}{2}$.

Drug off: Cells reach x=0 during a descending period of length $\frac{T}{2}$.

A plot is shown below for (arbitrarily selected) values of $t_{on} = t_{off}$ such that Case 2 holds.

In[15]:=
```
x[t_] := Piecewise[{{ω t , t < ton}, {1 - ω (t - ton) , ton ≤ t < 2 ton}}]
Plot[x[t] /. {ω → 1 / 5, ton → 5}, {t, 0, 10}]
```

Out[16]=

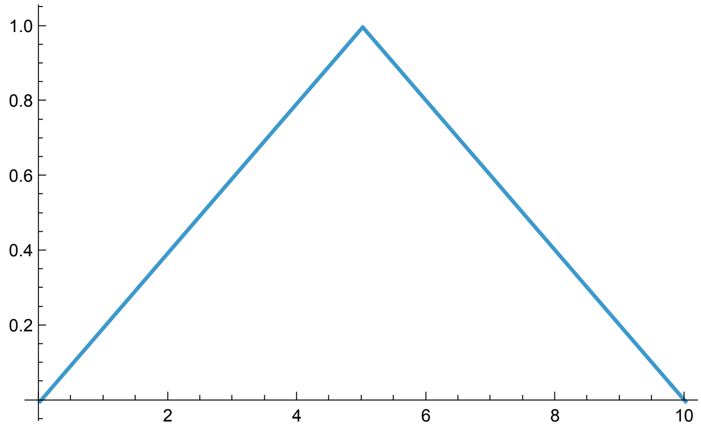

We now symbolically calculate and simplify the expression for the Case 4 integral.

In[17]:=
```
C4 = 1/(2 * ton) FullSimplify[
    Integrate[λ1[ω t], {t, 0, ton}] +
    Integrate[λ0[1 - ω (t - (ton))], {t, ton, 2 * ton}]
  ] /. {ton * ω → 1} /. {-FM0D - FM10 + 2 (FM0D + FM10) → FM0D + FM10 }
```

Out[17]=

$$\frac{1}{4}\,(\mathrm{FM00} + \mathrm{FM0D} + \mathrm{FM10} + \mathrm{FM1D})$$

We now consider Case 5 and 6 where $t_{on} = t_{off} = \mathrm{T}$.

Case 5: $T < \frac{1}{\omega}$.

Case 6: $T > \frac{1}{\omega}$,

In[18]:=
```
C5 = 1/(2 T) FullSimplify[
    Integrate[λ1[ω t], {t, 0, T}] +
    Integrate[λ0[ω T - ω (t - T)], {t, T, 2 T}]
  ] /. {-FM0D + FM1D → ΔFMD} /. {-FM00 + FM10 → ΔFM0}
```

Out[18]=

$$\frac{1}{4}\,(2\,(\mathrm{FM00} + \mathrm{FM0D}) + \mathrm{T}\,(\Delta\mathrm{FM0} + \Delta\mathrm{FMD})\,\omega)$$

In[19]:=
```
C6 = 1/(2 T) FullSimplify[
    Integrate[λ1[ω t], {t, 0, 1 / ω}] +
    Integrate[λ1[1], {t, 1 / ω, T}] +
    Integrate[λ0[1 - ω (t - T)], {t, T, T + 1 / ω}] +
    Integrate[λ0[0], {t, T + 1 / ω, 2 T}]
  ] /. {FM0D - FM1D → -ΔFMD} /. {-FM00 + FM10 → ΔFM0}
```

Out[19]=

$$\frac{(\mathrm{FM00} + \mathrm{FM1D})\,\mathrm{T} + \frac{\Delta\mathrm{FM0} - \Delta\mathrm{FMD}}{2\,\omega}}{2\,\mathrm{T}}$$

SM3: Model parameters used in the main article results figures

The model parameters used to generate the simulation results shown in the main article are listed in Supplementary Table 1.

panel	η_{on}	η_{off}	FM(0,0 nM)	FM(1,0 nM)	FM(0,300 nM)	FM(1,300 nM)	FM(0,500 nM)	FM(1,500 nM)	initial condition
**Figure 2**									
c; 300 nM; no update	-	-	0.137 day^{-1}	0.071 day^{-1}	-0.202 day^{-1}	0.171 day^{-1}	-	-	Figure 4 (day 1)
c; 300 nM; all other panels	2	2	0.137 day^{-1}	0.071 day^{-1}	-0.202 day^{-1}	0.171 day^{-1}	-	-	Figure 4 (day 1)
c; 500 nM; no update	-	-	0.137 day^{-1}	0.071 day^{-1}	-	-	−0.202 day^{-1}	0.171 day $^{-1}$	uniform
c; 500 nM; unbiased	1	3	0.137 day^{-1}	0.071 day^{-1}	-	-	−0.335 day^{-1}	0.146 day $^{-1}$	uniform
c,e; 500 nM; semi-biased	3	4	0.137 day^{-1}	0.071 day^{-1}	1	3	−0.268 day^{-1}	0.171 day $^{-1}$	left-shifted half normal
c,e; 500 nM; biased	-	-	0.137 day^{-1}	0.071 day^{-1}	-	-	−0.202 day^{-1}	0.171 day $^{-1}$	left-shifted half normal
**Figure 3**									
b; 300 nM	η	η	0.137 day^{-1}	0.071 day^{-1}	-0.202 day^{-1}	0.071 day^{-1}	-	-	Figure 3a
e; 300 nM	η	η	0.137 day^{-1}	0.071 day^{-1}	-0.202 day^{-1}	0.071 day^{-1}	-	-	Figure 3d
b,c; 500 nM	η	η	0.137 day^{-1}	0.071 day^{-1}	-	-	−0.287 day^{-1}	0.074 day^{-1}	Figure 3a
e,f; 500 nM	η	η	0.137 day^{-1}	0.071 day^{-1}	-	-	−0.287 day^{-1}	0.074 day^{-1}	Figure 3d
**Figure 4**									
no update	-	-	0.137 day^{-1}	0.071 day^{-1}	-0.202 day^{-1}	0.171 day^{-1}	-	-	in top figure row
all other panels	2	2	0.137 day^{-1}	0.071 day^{-1}	-0.202 day^{-1}	0.171 day^{-1}	-	-	in top figure row

Supplementary Table 1: Model parameters used to in the simulation results presented in the main article. The initial phenotype distribution (initial condition) is described in the right-most column.
